# Supplementary material for: IgG Antibody Responses to Epstein-Barr Virus in Myalgic Encephalomyelitis/Chronic Fatigue Syndrome: Their Effective Potential for Disease Diagnosis and Pathological Antigenic Mimicry
Source: Medicina (Kaunas). 2024 Jan 15;60(1):161. doi: 10.3390/medicina60010161 (PMC10820613; doi:10.3390/medicina60010161)
Supplement: Supplementary file 1 [file medicina-60-00161-s001.zip › medicina-2761006-supplementary.pdf]

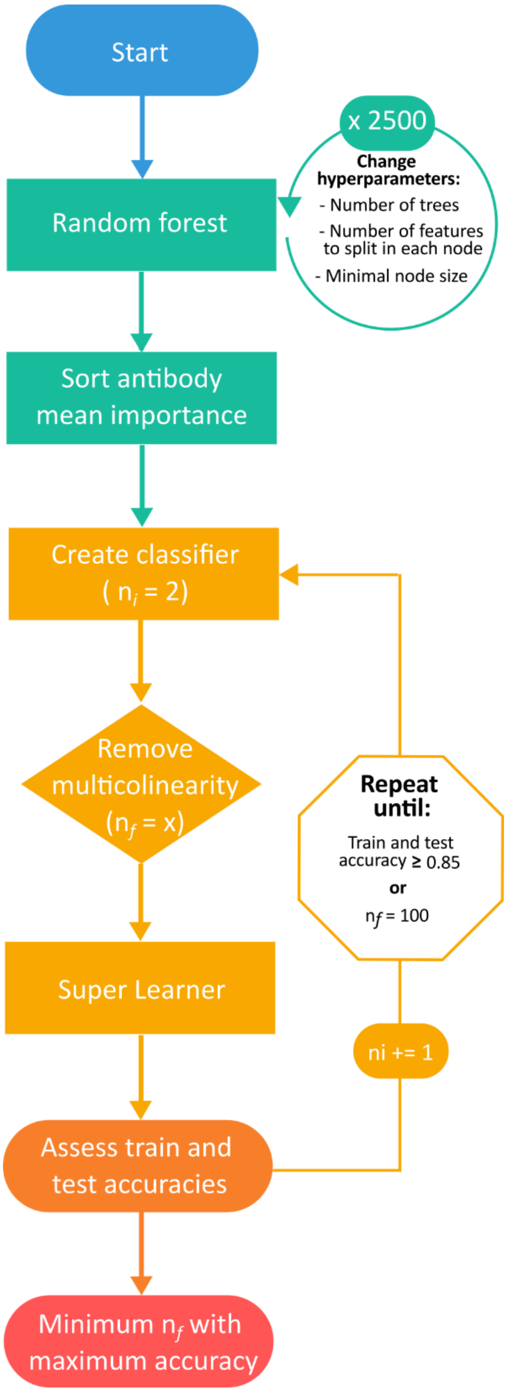

Figure S1. Pipeline for data analysis where different steps are shown in the flowchart using distinct coloured shapes. Green refers to the Random Forest step, where two-thousand and five-hundred Random Forest runs were conducted while changing the model hyperparameters. The average antibody importance was then obtained, and antibodies were sorted according to their average importance. Light orange refers to the steps concerning the classifier construction and assessment of its train and test predictive accuracies using the SuperLearner. The process of creating a classifier and assessing its predictive performance through the SuperLearner was repeated, each time adding a new feature, until the stopping criterium (octagonal shape) was verified. Once a new feature was added to the construct, the Spearman coefficient was obtained to remove highly correlated features. The flowchart ended by selecting the optimal classifier, the one with the minimum number of antibody responses (k_{min}), while simultaneously achieving a train and test accuracy above or equal to 0.85. If the accuracy of 0.85 was not met, the classifier with the minimum number of antibody responses and highest accuracy would be considered the best classifier.

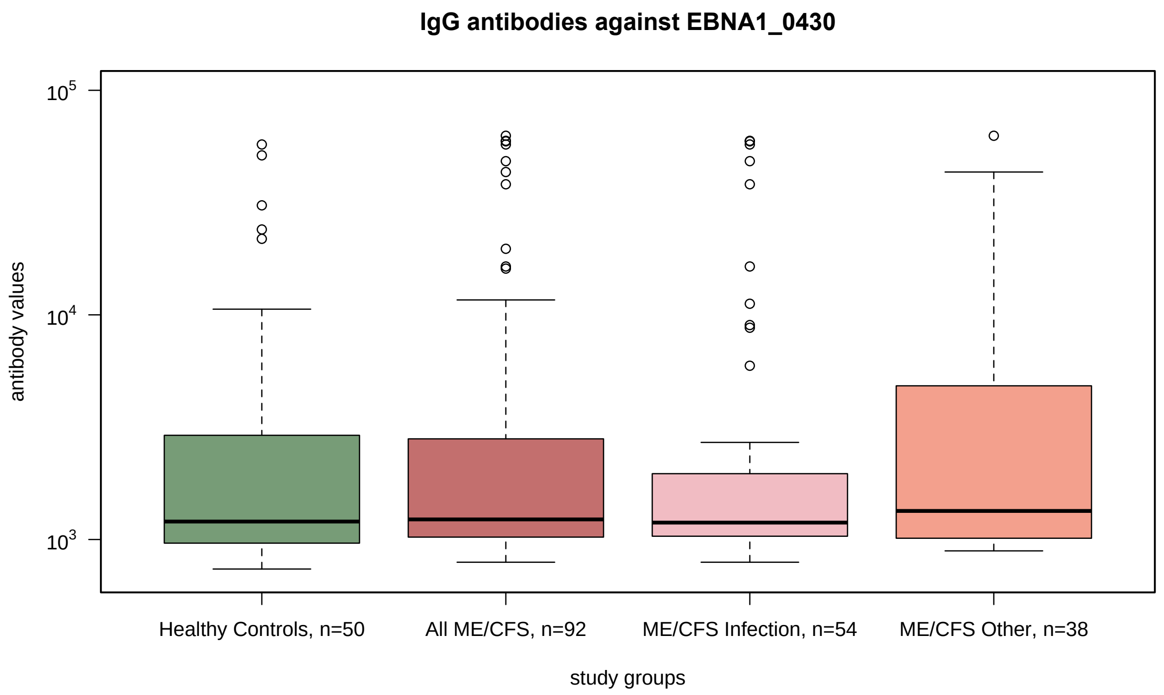

Figure S2. Boxplot of data related to IgG antibody against EBNA1_0430 in the whole ME/CFS group (All ME/CFS), the ME/CFS subgroup whose patients reported an infectious disease trigger (ME/CFS Infection), the ME/CFS subgroup whose patients reported a non-infectious disease trigger or did not know their disease trigger (ME/CFS Other), and their healthy controls. Differences in antibody distributions between ME/CFS group/subgroups and healthy controls were not statistically significant according to the Mann-Whitney-Wilcoxon test with continuity correction (All ME/CFS versus HC, p-value = 0.410; ME/CFS Infection versus HC, p-value = 0.626; ME/CFS Infection versus HC, p-value = 0.322).

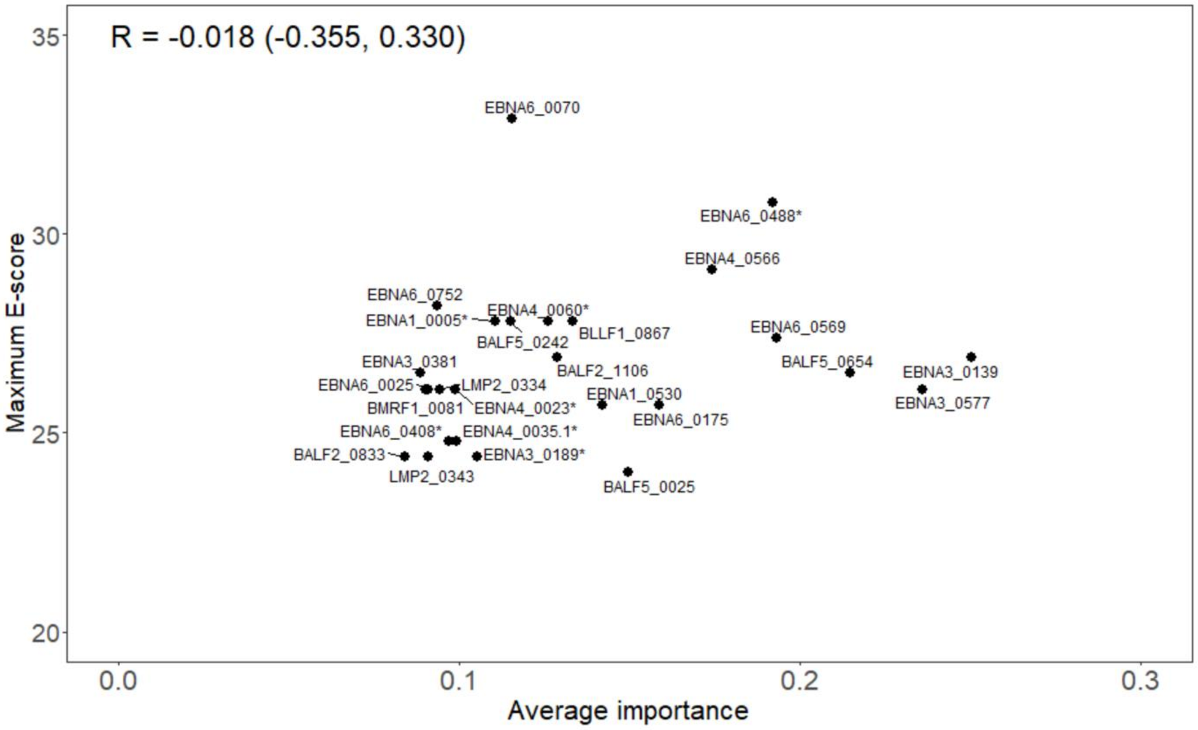

Figure S3. Scatterplot between the average importance of each EBV peptide associated with the 26 selected antibodies and the maximum E-score alignment score with human proteins using the nr protein database where R is the Spearman's correlation coefficient with the respective 95% confidence interval in brackets.
